# Dual-use, quantum technologies, and responsible innovation: the challenges of responsible quantum in defence

**DOI:** 10.1140/epjqt/s40507-026-00531-w

**Published:** 2026-06-16

**Authors:** Carolyn Ten Holter, Mira Wolf-Bauwens

**Affiliations:** 1 University of Oxford, Oxford, UK; 2 GESDA, Geneva, Switzerland

## Abstract

The development of novel technologies does not take place in a moral, economic, or geopolitical vacuum. Therefore, questions of funding, use-cases, and contexts of use will directly influence both moral and practical judgements around the ways in which these technologies are, or should be, developed and deployed. This article examines the growth of Quantum Technologies (QTs) in light of these challenges, and the ways in which they are already raising concerns with respect to questions of ‘dual-use’. The article argues that ‘dual-use’ as a category is inadequate for normative judgement as it conflates moral evaluation with a descriptive classification, and therefore misses important contextual and procedural elements. The article proposes making Responsible Innovation (RI) frameworks integral to the development of QTs in order to enable context-sensitive ethical assessment and decision-making guidance. Finally, the article proposes a way forward that engages with the principle-based concerns of those critical of military uses of QTs, while respecting the challenges posed by current geopolitical realities.

## Introduction

Global instability is not a new phenomenon, but the international order may become more or less stable over time depending on multiple variables. Such variables are frequently well understood at the global level, and the rules-based international order has broadly maintained an equilibrium for the last 80 years in response to such understanding.

However, this balance is easily affected by events, whether foreseen or not, and the development and rollout of novel technologies can be a disruptive element, especially when balances of power are affected. This is particularly true of technologies that have utility in both civilian and military spheres, known as ‘dual-use’. The history of technology is scattered with innovations that sprang directly from the exigencies of conflict. Although the world is not globally at war there is, at the time of writing, increasing competition and friction between nations, with ‘proxy’ wars playing out in various theatres. As technology becomes increasingly embedded in everyone’s lives, the civilian and military use-cases for technology may be closer than ever before.

An examination of such technologies must therefore acknowledge these considerations, and take account of the geopolitical background against which such technologies are developed. This article examines the development of Quantum Technologies (QTs) as one such dual-use set of capabilities, and considers some of the questions and challenges that should be investigated in this context. In particular, the paper investigates the often binary moral arguments that coalesce around dual-use technologies, and seeks to provide a more differentiated analysis. Looking at the main definitions of the phrase ‘dual-use’, one can differentiate between dual-use as a *descriptive*, value-neutral categorisation; dual-use as a *regulatory* concept, for example a categorisation used to justify export controls and IP rights; and dual-use as a *normative* framework. While the *descriptive* and the *regulatory* use have their own challenges which we will come across in this paper, our main focus is on the use of ‘dual-use’ as a normative category.

The article argues that ‘dual-use’ as a category is inadequate for normative judgement as it conflates moral evaluation with a descriptive classification, and therefore misses important contextual and procedural elements. It develops a way forward that draws on Responsible Innovation (RI) techniques to support ethical and responsible QT development in military and Defence use-cases.

### The origins of ‘dual-use technologies’

The earliest reference we could find for the specific term ‘dual-use’ appears in the literature in 1993:

*“Understanding the extent to which ‘dual-use’ technologies or products – those also having legitimate applications – are involved in the development of weapons of mass destruction is important, since both the feasibility of controlling dual-use items and the implications of doing so depend on the extent of their other applications”* [47]

It is clear from this definition that a very specific set of technologies was considered; those that can be used as components in, or as, weapons of mass destruction. However, the article does not define the term and it is also clear that the authors assume the concept of technologies and artefacts with both civilian and military use-cases to be well-established at this point. The National Research Council broadly concurred in 2004, *“In the language of arms control and disarmament, dual use refers to technologies intended for civilian application that can also be used for military purposes.”* – although in this case the question of the ‘legitimacy’ of the applications (and the equation of ‘legitimate’ with ‘non-military’) was not considered. [26]

Pustovit & Williams [31] define the term differently, avoiding questions of civilian or military domains and instead focusing on the impacts of the technology *“dual use technologies refers to research and technology with the potential both to yield valuable scientific knowledge and to be used for nefarious purposes with serious consequences for public health or the environment*” [31]. The use of the term ‘nefarious’ indicates the author’s own position on such technologies and picks up the implied theme of military use-cases being morally wrong.

The same year, Forge was delineating the ‘moral’ component of dual-use *“...a technology... has a... primary purpose which is good...and a secondary purpose ...which is bad... This is supposed to raise, or should raise, moral problems for researchers, for instance about responsibility.”* [10]. The clear assumption here is that the ‘good’ use is for civilian or public benefit and the ‘bad’ is for Defence or military purposes. The addition of the note about researcher responsibility is a theme we will discuss below.

The Law of Armed Conflict and the Geneva Conventions do not recognise dual-use objects [14], but clearly the broad concept of technologies that have both civilian and military application is much older than the current discussion. For example, bowmen in the medieval period both hunted and went to war, but as more advanced technologies have become more numerous and more prevalent, these challenges have become more frequent. The last century saw enormous technological leaps, and the intensity of conflict was clearly a driver in some instances. WWII alone accelerated the development of computers; radar-enhanced meteorology; skin grafting; mass production of penicillin; the atom bomb; ground-based navigation that led to GPS; blood plasma transfusion; and other scientific insights. However, the idea that the pressured atmosphere of conflict generates additional or more rapid creativity and engineering breakthroughs isn’t universally accepted. Sir Henry Tizard (Rector of Imperial College, London, 1929-1942) led on supporting the development of radar, as well as jet engines, atomic weapons and operational research in the 1930s. However, in 1948, when he was the equivalent of chief scientific advisor to the Ministry of Defence, he said: *“It is a mistake to suppose that science advances rapidly in a war. Certain branches of science may receive a special stimulus, but on the whole the advance of knowledge is slowed”* [42].

This paper challenges the assumption that the moral judgment of such technologies – assigning technologies and their uses to ‘good’ and ‘bad’ categories – is sustainable without taking account of use-cases, stakeholders and context. This context might include factors such as the broader geopolitical position; the situation of the state using the technology and the relative positions of its competitors; the impact of the technology (for example, if a technology may reduce civilian casualties, is its use by a military still to be deemed ‘bad’?); or even the realistic prospect of effectively separating use-cases – such as soldiers using their personal smartphones in professional contexts. In other words, it is our position that a technology *cannot be morally evaluated based simply on the locale or funding of its development*. The way in which it has been developed, and the purposes and context of use, are more relevant factors for making such a judgement.

Secondly, the paper proposes the incorporation of RI practices at each step of research, development, deployment and use. As ‘dual-use’ is merely a *descriptive* framework for potential applications, it does not provide normative guidance and therefore cannot provide guidance for ethical decision-making. RI fills this gap by moving the focus from categories of potential use to a more holistic analysis. Without such a framework – that can take account of stakeholder requirements and impacts, examine contextual use-cases, and incorporate anticipatory governance – we argue that it is not defensible to adopt a hardened position around whether it is ethical or unethical to use a particular technology for a particular purpose or in a particular context.

## Dual-use and QTs

While there is extensive literature on dual-use in general, the literature on QTs and dual-use is young, although not entirely lacking [19, 41, 51]. As with any technology that is both costly to develop and may have use in multiple domains, Defence funding is frequently part of the investment supporting research, and QTs are of significant interest at the nation-state level. QTs are maturing and becoming commercialised, and their status as national-level investments means they are therefore increasingly affected by geopolitical tensions, and increasingly scrutinised. This also has implications for the security of the research, as increased geopolitical tensions can mean technologies such as this receive more attention from competitor states [51]. Walker-Munro [51] and others also point out that the second wave of quantum has a “fundamentally disruptive capacity” (Walker-Munro [51], p2) as it transmutes existing technologies [20]. The political context for the current level of global investment in QTs is the first war in Europe since the 1990s; the changing nature of warfare driven by the Russia-Ukraine conflict, which has seen a huge acceleration in the use of AI for military purposes; and increasing attacks on international law and the rules-based order. Therefore, careful assessment is needed of the way in which technologies such as quantum – that may have an impact on the geopolitical balance – are developed and utilised. In order to better understand the concerns around QTs and dual use, this section discusses different contexts through which the relationship between QTs and Defence use is problematised.

### Visible concerns: QTs outside of the laboratory

**Figure 1:**
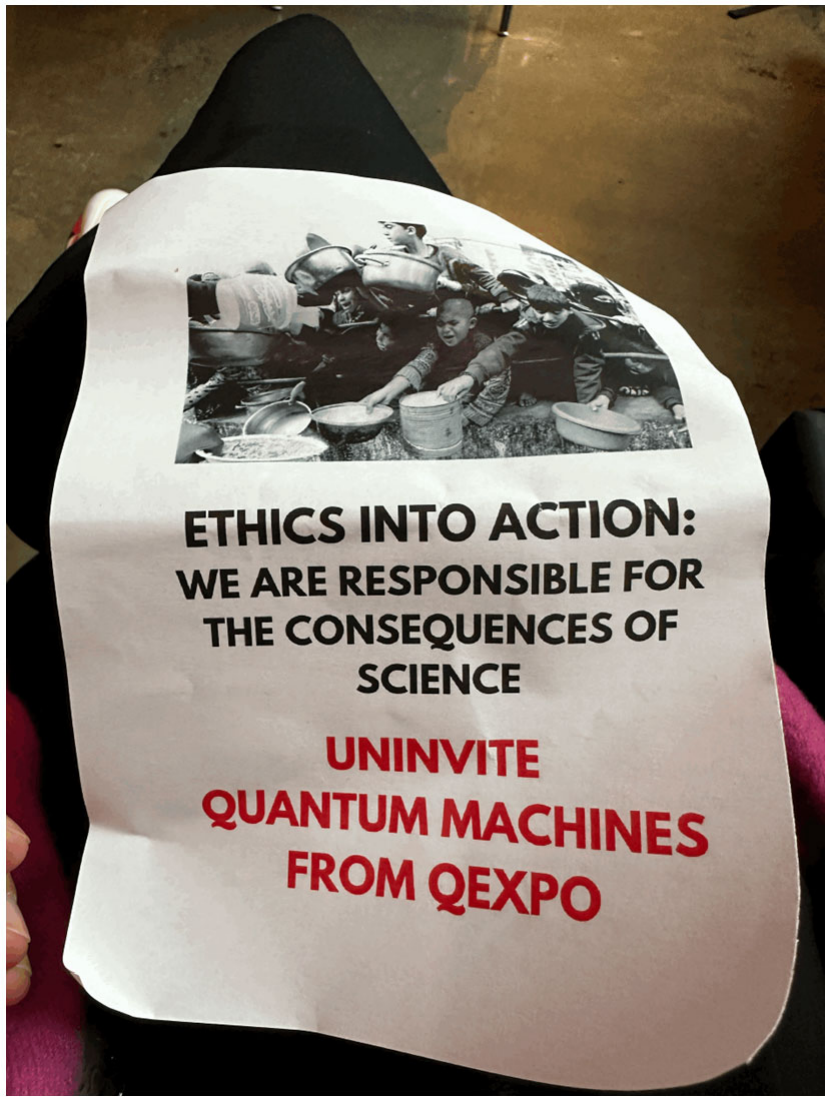
Leaflet from the protestors at QExpo

In addition to commentators calling for greater ethical awareness (eg Taddeo et al. [41]), there are already visible tensions stemming from an awareness of the current political realities, motivation(s) for funding QTs and the possible effects on QTs. At a major European industry conference in 2025 (QExpo, held in May 2025 in Amsterdam) a small protest was staged against a quantum computing company involved in building a quantum computer for the Israeli Defence Force. Researchers called for a boycott of the conference as the company concerned had a booth in the main conference hall. Leaflets distributed at the entrance of the conference read “*ETHICS INTO ACTION: We are responsible for the consequences of science*” (See: Fig. 1).

There are also signs of disquiet in other quarters of the quantum community – the “Disarm Quantum” group has launched a manifesto that calls for signatures and *“reject[s] the use of our research for military applications”*. There is obvious alarm from these scientists that their work may be used for Defence or military purposes, and this is likely to be exacerbated by what they see as an absence of ethical or responsible consideration of these purposes. The group also expresses unease around the funding of quantum research, calls for the cessation of Defence funding, insists on the neutrality of academia, and deplores what it terms the ‘race for rearmament’.

Real-world challenges such as these make it clear that there are already significant concerns around dual-use in QTs. As QTs approach higher TRL levels, both potential and actual use-cases for military and Defence become clearer. The development of quantum computing in particular is being closely monitored by agencies and institutions worldwide. In the UK these include bodies such as the National CyberSecurity Centre, GCHQ, the Ministry of Defence, the Centre for the Protection of National Infrastructure and others. In the US there is involvement from the FBI, the National Institute for Science and Technology, and targeted bodies such as the National Counterintelligence Task Force’s (NCITF) Quantum Information Science Counterintelligence Protection Team (QISCPT) [9]. This protective posture is highly likely to be reflected around the globe. Quantum computing’s profile as a research area characterised as important for technological sovereignty – and the attendant concern to become less reliant on US- and China-based tech – makes it one of only a handful of ‘enabling’ technologies seen as crucial at the national level (see Sect. 3.1). Those working in the field will be aware of this interest and can anticipate the effect that it may have on questions around funding streams in the future, ability to publish (and where), hiring practices, and inward investment.

However, it should be pointed out that the QT use-cases currently available do not have what might be termed an ‘aggressive’ position. These technologies include the MEG brain scanner designed to assess possible brain-damage to service personnel from blast exposure [4], or the quantum Position, Navigation, Timing (PNT) technologies that allow for navigation despite GPS-jamming [46]. The level of variation already being seen in use-cases and context for QTs makes it clear that an appreciation of the type, use, and context of the technology is required before moral or ethical judgement can be made. Simplistic or dualistic positions (civilian ‘good’; military ‘bad’, per Forge [10]) therefore become not only untenable, but morally complicated in and of themselves - for example would signatories to the Disarm Quantum manifesto wish to argue that service personnel should not have access to the most effective brain-scanning technology?

These examples make it clear why moral judgements based on the categorisation of ‘dual-use’ are insufficient.

#### Underlying values of technology

There is an extensive and deep-rooted philosophical debate around the question of whether technology is value-neutral [52], with arguments around intention (eg Heyndels [15]) frequently set against arguments around purposes (eg Fry [11]). However, this article argues that normative assessments of technology can only be made in their context of use. Analysing the context of use involves many elements: understanding the users; the intended use; and the direct and indirect stakeholders involved. It is also important to understand that normative assessments can differ along the value chain or the process of use of a technology. While there might not be a normative issue with a given QT while it is being developed in a lab funded by a national government science grant, if the technology is acquired by a Defence company, that judgement may alter. In today’s geopolitical realities, it can also not necessarily be argued that *any* military use is to be evaluated as negative. For example if the use-case is to safeguard civilian cybersecurity infrastructure, which in turn allows for preserving data privacy, then safeguarding military cybersecurity infrastructure is necessarily also included.

Hence, we argue that a more structured method of balancing these many factors is required, and propose that a more productive way to approach questions of QTs and ‘dual-use’ is from an RI perspective. As has been argued elsewhere, [20, 43, 44] and as we will lay out in the second part of this paper, RI can provide us with the tools to assess each stage of the QTs value chain and – if necessary – take action to ensure responsible use. The realities of the state of the technology and the limits of categorising something as ‘dual-use’ make it even more pertinent to understand why such a binary is insufficient to enable normative judgement or action-oriented decision-making.

Before discussing what an RI approach would entail, let us first understand how deeply intertwined QTs and the military sector are, in order to better understand why reflexive critical judgement towards QTs are often made so quickly.

### The military context and QTs

To understand the origins of concerns regarding QTs and dual use, it is helpful to understand the potential applications of QTs in the military context. Generally speaking, the three main QTs (quantum sensing, quantum computing and quantum communication) have different use-cases. These include the development of new molecular structures for novel materials, or optimisation of complex planning (quantum computing); the unprecedentedly precise measurement of physical properties, or the ability to see through smoke and fog (quantum sensing); and secure communications (quantum communication) for the transmission of data [8]. While each of the technologies also has apparent civilian uses - from sustainable materials discovery to better medical diagnosis - they also have clear potential uses in the military domain. These have been covered at length in Krelina [23], Gallego Torromé & Barzanjeh [12], Karsa et al. [21] and others. We recommend the reader interested in concrete examples to examine these works for more detail.

#### Military funding of QTs

In addition to specific use-cases for QTs in military contexts, it is also important to consider the close connection between QTs and the sources of development funding (this is also recognised in the Disarm Quantum Manifesto). Historically, quantum research has been closely connected to military and Defence funding streams [23]. It has also been argued that Defence priorities are a significant factor in shaping national quantum strategies [29]. This is seen explicitly in concepts such as the ‘Fusion Doctrine’ – a precept developed in the UK that describes an area where ‘security’ and ‘prosperity’ concerns are melded and therefore require a holistic perspective [25]. The result often is a ‘whole of government’ approach that does not differentiate between Defence and other research spending. The connection between security and prosperity for some of these technologies, including quantum and AI, is demonstrated by the perception that countries need to invest heavily to remain ‘in the race’ [44]. This has implications both for investment and for governance, as the Fusion Doctrine explicitly recognises that – both in terms of national security and national economic interests – government has many possible routes to its desired ends. The doctrine is not a policy in and of itself, but it builds this approach of the melded security and prosperity aspects into all other assumptions at national level.

Elsewhere, the European Commission highlights key use-cases of different QT in Defence include “position, navigation, and timing (PNT) applications, such as inertial navigation in GNSS-denied environments, inertial navigation for autonomous vehicles, atomic clocks, quantum imaging and radar, as well as optronics and secure communications.” As a result, the European Defence Fund (EDF) is supporting the development of QT for use in Defence contexts with an investment of EUR60million [8].

In the US, the Defense Advanced Research Projects Agency (DARPA) has announced a quantum-benchmarking initiative (aimed at verifying which quantum computing hardware platform can achieve utility-scale operations by 2033), and has committed to USD1 billion of funding [7]. The majority of leading quantum computing companies are receiving funding as part of this project. At the same time, DARPA also provides matched funding for regional quantum hubs in Illinois, Maryland and New Mexico - DARPA has been funding quantum research since the 1990s, and as early as 2001 launched one of the first comprehensive programmes to study quantum computing and its uses [6]. Based on these early and significant investments, DARPA states that it is now seeking to *“exploit emerging quantum processing technologies for national security.”*

It is clear that Defence or Defence-adjacent funding, connected to military purposes, is a prominent driver of the research and development of QTs – if not among the key drivers. This must be taken into account when considering the ways in which more nuanced approaches can support ethical assessments and responsible development of technology.

However, it should also be noted that where this funding is linked to government, it will be constrained in important ways by oversight, and scrutiny and reporting requirements. Even funding sourced from Defence Primes, who (at the time of writing) are not heavily engaged in quantum research, would need to align with government and national priorities and policy concerns. The engagement of national governments in such funding, as has been discussed elsewhere [44], offers significant levers for the inclusion of RI and other governance techniques. This sets QTs apart from some other powerful technologies, such as AI, that do not afford such levers.

#### Directionality

It may also be worth understanding whether present concerns with dual-use technologies may be influenced by what might be termed ‘directionality’. This may mean, for example, that the microwave - developed from the cavity magnetrons used to amplify radar signals in WWII - is seen as a useful, unexceptional addition to the kitchen, but that the technique enabling aerosolisation of a gypsy-moth-control bacterium potentially being used to provide an aerosol of anthrax is deeply problematic [5]. Military technology providing benefits to civilian populations (eg GPS) may be more acceptable than civilian technology (and many would argue QTs fall into this category) being used for military purposes. It is possible that this ‘directionality’ is important in shaping public responses to technologies that can be used in both military and civilian spheres. However, literature searches have not revealed any examples of public attitudes to dual-use technology so it is not possible, at this point, to go beyond speculation as to whether this ‘directionality’ would be an important consideration.

Additionally, in the context of QTs and the historical context during which they are being developed, we may observe that ‘directionality’ is less evident in the current moment, as civilian and Defence use-cases may occur simultaneously. While recent significant investments into QTs, such as the Quantum Benchmarking Initiative [7], seem to be in line with the desire to use QTs for national security, this does not prevent use that would be of wider societal significance. For instance, knowing which quantum hardware platform is the most promising to achieve quantum utility (and eventually advantage) will permit of more concerted efforts into developing this hardware platform to a point where it can be used for drug discovery or the simulation of materials for carbon capture. This would thus be a case where the directionality of the dual-use is such that the origins are in the Defence domain but the potential benefits might be more widespread. Those who are critical of the use of QTs in the military domain, and Defence sources of funding, may regard this development in a different light.

## Geopolitical considerations

Having discussed the most evident dual-use contexts for QTs, we turn to the wider geopolitical arena, as it further highlights the extent to which a nuanced and differentiated analysis is a necessary prerequisite for the development of a normative evaluation. We show that geopolitics does not merely add another context of use but offers a fast-changing background against which we need to evaluate the distribution of risks, benefits, key stakeholders and their responsibilities. Put differently, current geopolitics requires that we resist simplified moral judgements.

### QTs as strategic and sovereign assets

As we have briefly discussed above, QTs are regarded as of nation-state interest, and may (such as in the UK’s ‘Fusion Doctrine’) be viewed as important for both security and economic reasons. In this context they are increasingly framed as strategic assets [23, 49]. Many countries consider QTs to be a matter of sovereign capability, and crucial for technological sovereignty (eg HM Government[16]). At the international level, this has already had an effect, despite the early TRL of most QTs. In the US and UK, regulations such as export controls have already been tightened [53]. Frameworks such as ‘trusted research’ in the UK [28] now require consideration of questions such as national intellectual property - as a result, international collaboration and research has been made more difficult. The UK’s National Security and Investment (NSI) Act 2021 means that investments in some technologies, or from certain countries, or over a certain percentage of equity, are automatically required to be reported. It also allows the relevant secretary of state to ‘call in’ an investment in a UK company for inspection, and potentially require it to be repudiated, up to five years from the initial investment, demonstrating the degree of concern about these ‘future’ technologies.

**Figure 2:**
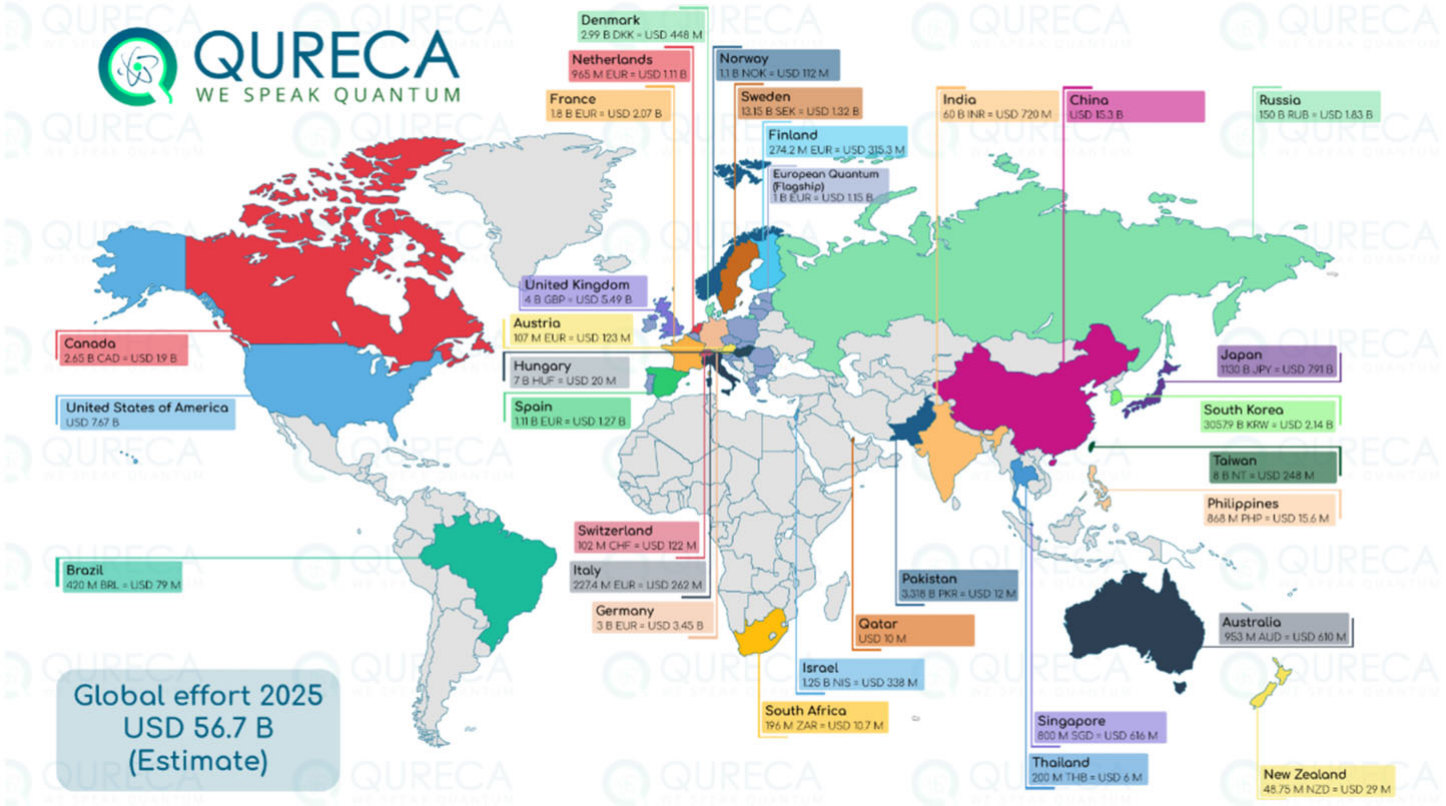
Global quantum investment as at 2025

The global map of quantum investment (Fig. 2) demonstrates the significant funding that has been ringfenced for QTs in recent years (Source: Qureca). The number of countries with a national quantum programme has expanded rapidly over the last decade, as have the sums involved. The Quantum Economic Development Consortium, which tracks quantum investment and the state of the international quantum economy, saw unprecedented growth in 2025, with patents, venture capital investment and government support all increasing rapidly. The most recent report gave a figure of a 310% increase in US govt spending during 2025 [18]. Nation-states are not investing in this way to progress foundational research – commentators argue that governments are investing heavily to support technological development seen as vital both for economic and national security. [13] This is partly driven by Shor’s algorithm – an algorithm that, if run on a sufficiently powerful quantum computer, could decrypt most of today’s classical encryption. As we will discuss below, this algorithm has led to significant concerns around cybersecurity, driving research into quantum computing and, as a side-effect, drawing funding into research of other QTs. Such security concerns are of global interest, and clearly cybersecurity is both a civilian and Defence concern. This underlines how - as soon as we start to investigate quantum computing - questions of dual-use and national security come to the fore. “Sovereign capability” technologies are those deemed to be so important that it is vital a country has access to its own version [16]. So questions of sovereign capability are central to the assumptions that go into developing policy for novel technologies, but there are other factors as well. It is argued that these concerns and discourses are used to legitimise sovereignty-first policies: *“The legitimising role of this discourse is particularly clear in the case of QTs, which are more broadly understood to belong to the future.”* (Vogiatzoglou & van Hoboken [49], p. 27).

### QTs and security

#### National security beyond military contexts

National security in the modern world is necessarily considered as a holistic, multi-factoral issue. These factors are not limited to Defence and the military - in an era of globalisation, national security considerations involve inward investment, infrastructure, immigration, cybersecurity, supply-chains, food and economic security, and climate impacts. This thinking can be seen, for example, in both Europe and America investing in domestic chip production in order to not have to rely on imported chips for industry [36].

The international stage and its relationships are key in other ways too. These types of relationships - including soft power, the complex geopolitics of Defence, and global technological leadership - are important threads in discussions about dual use. Already this analysis shows that the connection between geopolitics and QTs is of such complexity that the use of binary analytical tools such as the ‘dual-use’ paradigm has significant limitations. This will become further evident in the Sect. 3.2.2 where we discuss the connection between QTs and security.

#### Cybersecurity

As is now well-known, the threat to global cybersecurity represented by Shor’s algorithm is probably the most prominent example of a use-case for a quantum computer [39]. The algorithm is designed to allow a quantum computer to factorise the large numbers used in most of today’s encryption – it therefore poses significant risks [35]. Cybersecurity is a field that concerns both Defence and civilian domains. Therefore, considering this risk as solely civilian or solely military would be a risk in itself. The recognition that these concerns apply across all domains has so far prevented the risk, and any developing technologies that meet it, being categorised as single-domain. For instance, if considering it military-only, possible defence mechanisms might be embargoed initially and would only make it into the civilian realm late – possibly too late. In this case, the US National Institute of Standards (NIST) ran an open competition in search of novel cryptographic algorithms that would withstand Shor’s algorithm [27]. The eight-year competition resulted in four algorithms being chosen for standardisation. These algorithms are open source and hence can (and should) be used by any organisation – be it military or civilian – to ensure their cryptography is quantum-safe.

The cybersecurity context is another area that illustrates that moral judgement of a technology based on the context of use alone is too simplistic. Returning to the ‘Disarm Quantum’ manifesto, it seems both unlikely and impractical that Defence organisations should be prohibited from operating cyber-secure systems. This is particularly important given the geopolitical reality that today’s wars are increasingly conducted using online and internet-based technologies in a “hybrid” or “non-linear” manner [17, 34].

Considered together, these dynamics shows that geopolitics does not merely add a context of use for QTs but actively structures how and why QTs are funded, governed, restricted, and legitimised. This further exposes the limitations of a dual-use judgements that rely on binary distinctions. It reinforces the need for a more differentiated normative evaluation of QTs.

## Beyond ‘dual-use’

There is clearly (and, in healthy democracies, should be) a spectrum of views around the ethical status of dual-use technologies in general and QTs in particular. Some commentators adopt an extremely robust position on principle, reflecting the “civilian:good, military:bad” approach of Forge [10] discussed in 1.2 above. For example, McKay [24] frames the US approach to these questions as an ‘imperial’ one, describing the US and militaries in general as imperialist. McKay warns that militaries are already interested in and probably using QTs, and considers ways to prevent this - even questioning in another forum whether QTs should be pursued at all.^1^

Others take a warier or more pragmatic view, regarding the geopolitical competitive or adversarial threat to be significant enough that opportunities either to increase a ‘home’ advantage or to increase public safety should not (must not) be ignored. An example of this is the Global Positioning System (GPS) signal; crucial for tracking, location, mapping, timing, and navigation. This can be jammed or blocked, as indeed has been happening with increasing frequency in recent years. During 2025 Sweden recorded 733^2^ incidents of GPS interference in the Baltic Sea area during the year to September, with obvious significance for civilian aircraft and other use-cases. It does not seem reasonable to argue that if a quantum-based technology can supplant GPS-based Positioning, Navigation, and Timing (PNT) devices for civilian purposes, that the military should be barred from using it on grounds of principle. Indeed, the military willingness and ability to work with researchers to advance development of quantum PNT may have accelerated civilian access to a technology that will prevent needless accidents caused by GPS interference.

We argue that ‘dual-use’ should therefore not be used as a normative category. As a descriptive term, it may have utility for regulatory reasons such as export control regimes, but even there it is problematic. Since we argue that classifying something as military does not mean it is ‘ethically bad’ or as civilian and ‘ethically good’, the categorisation does not serve a purpose beyond categorisation itself. Instead, it would be more productive to look at more differentiated classifications that do away with simplistic dualities. An alternative is to understand dual-use as a strategy [22]. This perspective reflects how private companies understand different market segments for their products and can consider “dual-use” as a *“market strategy that might be deployed defense-first, commercial-first, or both”* [22]. This also applies outside the commercial setting, such as at universities. Walker-Munro argues that due to the proliferation of “dual-use” definitions, interpretations and governance structures, the conception loses its meaning and is insufficient to enable meaningful governance or actions [51].

It is also important to remember that questions around dual-use are not novel. In this instance lessons can be drawn from AI governance, where the absence of anticipatory governance and oversight in advance and throughout deployment has left governance and regulatory bodies scrambling with prohibition and control mechanisms. Blanchard et al. [1] agree, discussing the importance of ethical oversight and anticipatory governing for emerging technologies. In particular, they propose five principles drawn from AI that they believe are generalisable to the quantum domain: *“(1) an information-security-centric approach; (2) justified and balanced securitisation; (3) multilateral collaboration and oversight; (4) promotion of fusion development strategies for societal benefit and peaceful applications; and (5) a long-term strategic vision to counter authoritarian uses of quantum technology”* (Blanchard et al. [1], p. 5).

We suggest that although ethics-based approaches, such as those set out by Blanchard et al. [1] and Taddeo et al. [41], are important, they lack concrete methods through which to apply such principles. Additionally, focusing solely on ethics can create challenges of its own – such as ethics-washing - as Shelley-Egan [38] points out. Knowing that a technology can be used for both civilian and military purposes tells us nothing about whether it ought to be developed, funded, or deployed in any particular context. As “dual-use” provides no guidance for ethical decision-making, we need to go beyond the term. Similarly, consideration of ethics does not give us actionable information. For that, we need a framework attentive to process, stakeholders, and context. Therefore, we argue that as we look to move beyond dual-use as a way of framing ethical judgements around the uses of QTs, ethical principles such as these need to be extended to incorporate RI approaches. RI provides a framework with a more robust and actionable basis, and allows focusing on governance, engagement, accountability, and societal benefit.

### RI for quantum in defence

As discussed above, it is clear that the conceptualisation of a technology as dual-use fails on several fronts. Firstly, its descriptive purpose is oversimplified; it imposes a superficial binary on technologies that often have multiple, intersectional applications across civilian and Defence domains. Secondly, it is normatively empty; classifying a technology as dual-use does not allow us to come to any conclusions about whether or not its use is ethically permissible. Thirdly, trying to fit a technology – and in particular QTs – into a “military” or “civilian” category is not only difficult but often does not serve any purpose. By knowing only the use-case, without context, intent, constraints (eg regulation), available actions, or stakeholders, we know too little to enable the formation of a normative judgement. Fourthly, even the definition of ‘dual-use’ is contested. Finally, the concept is not actionable – it does not allow us to draw recommendations for actions from the categorisation.

We argue that what is required is a set of concrete actions and sources of insight, in a holistic framework that supports decision making and governance. Focussing on process and context can enable a more nuanced normative assessment and open the possibility for stakeholder engagement mechanisms and anticipatory governance to be integrated in said process. RI allows for anticipatory work that does not seek to pre-judge technologies or their use-cases on the basis of domain, or funding background, but rather seeks to ensure that relevant consultation, consideration of impacts, and the ability to develop measured responses, will be more useful in the longer term.

It is in these respects that RI appears particularly apt for a technology in the process of moving from the research to the commercial sector. Although definitions vary, one of the most useful comes from the European RRI-Tools project:^3^

*“RRI is a way to do research that takes a long-term perspective on the type of world in which we want to live…[RRI means] involving society in science and innovation ‘very upstream’ in the processes of R&I to align its outcomes with the values of society.”* (www.rri-tools.eu)

RI methodologies therefore draw together multiple threads. They incorporate consultation with those who may be affected; consideration of both negative and positive outcomes; and responding to both these consultations and reflections – potentially influencing the course of a technology if deemed necessary [50]. RI is thus of necessity a highly interdisciplinary field, requiring as it does an understanding of science engagement, governance techniques, and stakeholder participation. In the UK it is most usually actioned as the AREA framework [40], incorporating the lenses of Anticipation, Reflection, Engagement and Action. It emphasises an iterative approach, with concrete applicability and tools already in the public, industry, and policy domains [30]. Additionally, there is already a substantive body of work on RI in quantum (eg Coenen & Grunwald [2]; Vermaas [48]), that can turn its focus to the Defence sector in a timely and informed way. Finally, it is built upon a foundation of ethics, with an understanding that collaboration and practical application are of more relevance than broad principles [33].

An RI approach enables us to shift the question from “what category?”, or “good or bad?” to *“how should decisions be made, by whom, and with what accountability?”*

#### (Epistemic) access and power imbalances

A further area where RI can support normative judgement is in its consideration of societal impact. For example, this allows it to draw attention to structural issues such as the digital divide between global North and South and to recognise barriers to participation in the benefits of a technology [45]. These issues of access are another nuance to the geopolitical context – an RI approach allows us to ask who has access to the technology, and to knowledge about it, and the power that comes with such knowledge (Oughton et al, *forthcoming*). Existing power asymmetries are already being manifested in the quantum domain. As Seskir et al. [37] point out, this is problematic if the stated end-goal is democratising access to emerging technologies in order to share the benefits globally. Such challenges are also relevant from the Defence perspective. Not having access to a technology or to knowledge about it can, for example, reduce the ability or the perceived need to prepare defences against possible cybersecurity attacks. Cybersecurity is a global Defence concern, and an RI approach allows us to understand that to pay attention only to global North concerns would be shortsighted in the extreme.

## The way forward – a call for nuance

### Recognising the limits of category-based moral judgements

We have argued for a departure from a rigid binary classification of ‘civilian’ or ‘military’ and attendant ‘good v bad’ moral conclusions. We consider that such a departure frees critical governance processes from artificial pre-judgments. Not only do such pre-judgements prevent more nuanced discussion, but they do not align with realistic understandings of the interconnectedness of civilian and military domains; do not recognise the back-and-forth nature of Defence and government funding; do not appreciate the value of retaining government/policy involvement; do not allow citizens or publics to have a voice in the discussion; and do not recognise the pressure of geopolitical realities.

As this paper has demonstrated, there are numerous overlapping concerns around the practical, moral/ethical, functional, and economic impacts of the use of QTs in military and Defence spheres. It has also been argued that moral judgements cannot be presupposed at the outset. Regardless of whether a technology is classified as military or civilian, such a classification does not provide a robust enough foundation for a moral judgement and/or a ban on the use in a given context of a given technology.

The paper has also demonstrated that it is excessively simplistic to make a moral judgement based only on the origins of funding. In addition, as both research and the Defence sector are ultimately largely government funded, the case for attempting to keep these entirely separate is difficult to make but must be subject to debate and discussion in a democratic society.^4^ We have argued that integrating RI practices into the funding, research, development and deployment of these nascent technologies, would be a first step towards enabling such debate.

We suggest that in order to ensure responsible use and deployment of QTs, researchers, product developers, and policymakers require mature, open discourse, and nuanced perspectives. The avoidance of snap moral judgements also opens the way for procedural approaches such as RI that can broaden decision-making processes and integrate them into political and practical realities that incorporate considerations around context of use. RI has already been shown to be well-suited as a practical framework for embedding ethical reflection into quantum computing research, emphasising anticipation, reflexivity, inclusion, and responsiveness [43].

Importantly, integrating RI into the research, development and deployment of QT would enable a real change in technology governance. Instead of having to retrospectively govern undesirable consequences of the use of a technology, RI enables proactive integration of responsibility into the design, funding, and deployment stages of QTs [32]. Integrating RI practices into all stages of the process allows seeing potentially problematic issues (such as types of use, lack of diversified stakeholder engagement, power imbalances) much earlier on. In addition, the hope would be that it would avoid “ethicalisation” of emerging technologies [38]. RI approaches allow us to focus on structural issues such as militarisation of research institutions through funding, or inequality.

### Areas for further research

In calling for more nuance, we have also uncovered challenges that would benefit from further research to substantiate a more nuanced analysis. For example, we considered whether the ‘directionality’ of dual use has an impact on public perception, or whether publics have views around dual-use at all.

Another area to explore is whether RI approaches would have to be adapted to be more suitable for technologies that are of high relevance for the military and civilian context.

Some might argue that calling for further nuance makes taking decisions e.g. such as funding and investment decisions harder. We would argue that this is precisely why an integrated RI approach is required. It is not about making the decision easier but about increasing the quality of decisions and eventually allowing for a democratic discourse responsible use of QTs.

### Conclusions

In this article we have argued that the ‘dual-use’ binary, while potentially useful for regulatory purposes such as export control, is fundamentally unsuited as a basis for normative judgement, and certainly cannot provide any moral certainties. The suggested dichotomy between civilian and military obscures rather than illuminates the ethical stakes of emerging technologies like QTs. Hence, we have proposed RI as an alternative that 1) acknowledges the complexity of real-world technology governance, 2) provides procedural mechanisms for inclusive deliberation, and 3) enables context-sensitive ethical assessment. In short, this is not a call to abandon moral judgement, but to ground it in processes not categories, and ensure that democratic voices have a place in the discussion around both the civilian and military technologies of the future.

## Data Availability

No datasets were generated or analysed during the current study.

## Footnotes

1 https://www.aps.org/apsnews/2022/08/should-we-build-quantum-computers.

2 https://www.bbc.co.uk/news/articles/clyx3ly54veo.

3 This is sometimes framed - was the case with this project - as Responsible *Research and* Innovation, but given the inclusion here of the commercial sector as well as the academic, this article uses the broader term Responsible Innovation.

4 It should be noted that QTs are not special with respect to this as Coveri et al [3] highlight in their article analysing the connection between “Big Tech” and the Military-Industrial Complex.

## References

[1] Blanchard A, Pundyk K, Taddeo M. Anticipatory Ethical Governance for the Research and Development of Quantum-enabled Defence Technologies (SSRN Scholarly Paper No. 4995497). Social Science Research Network. 2024. 10.2139/ssrn.4995497.

[2] Coenen C, Grunwald A. Responsible research and innovation (RRI) in quantum technology. Ethics Inf Technol. 2017;19(4):277–94. 10.1007/s10676-017-9432-6.

[3] Coveri A, Cozza C, Guarascio D. Big tech and the US digital-military-industrial complex. Intereconomics. 2025;60(2):81–7. 10.2478/ie-2025-0017.

[4] CSOC. World’s first mobile quantum brain scanner being developed to measure blast effects on troops. GOV.UK. 2025. https://www.gov.uk/government/news/worlds-first-mobile-quantum-brain-scanner-being-developed-to-measure-blast-effects-on-troops.

[5] Dando M. Biotechnology, weapons and humanity II. British Medical Association; 2004. https://www.abebooks.com/9780954861506/Biotechnology-Weapons-Humanity-II-Malcolm-0954861507/plp.

[6] DARPA. Quantum sensing and computing. 2020. https://www.darpa.mil/news/features/quantum-sensing-computing.

[7] DARPA. QBI: Quantum Benchmarking Initiative. 2025. https://www.darpa.mil/research/programs/quantum-benchmarking-initiative?utm_source=chatgpt.com.

[8] European Commission. Quantum Technologies in Defence. Publication Office of the European Union. 2025. https://defence-industry-space.ec.europa.eu/document/download/a09e0f8f-6f57-4d73-8ab0-b108fa840204_en?filename=Factsheet-Quantum-in-Defence_0.pdf.

[9] FBI. Protecting Quantum Science and Technology. 2024. https://www.fbi.gov/news/stories/protecting-quantum-science-and-technology.

[10] Forge J. A note on the definition of “dual use”. Sci Eng Ethics. 2010;16(1):111–8. 10.1007/s11948-009-9159-9. 19685170 10.1007/s11948-009-9159-9

[11] Fry H. Hello world: how to be human in the age of the machine. Transworld; 2018.

[12] Gallego Torromé R, Barzanjeh S. Advances in quantum radar and quantum LiDAR. Prog Quantum Electron. 2024;93:100497. 10.1016/j.pquantelec.2023.100497.

[13] Goorney SR, Aslan E, Baskakovs A, Muñoz B, Sherson J. National Quantum Strategies: a Data-Driven Approach to Understanding the Quantum Ecosystem. 2026. 10.48550/arXiv.2601.16329. arXiv:2601.16329.

[14] Hathaway OA, Khan A, Revkin MR. The dangerous rise of dual-use objects in war. Yale J Law Technol. 2024;134(8):2645–750.

[15] Heyndels S. Technology and neutrality. Philos Technol. 2023;36(4):75. 10.1007/s13347-023-00672-1.

[16] HM Government. Integrated review refresh 2023: responding to a more contested and volatile world. HM Government. 2023.

[17] Hoffman FG. Conflict in the 21st century: the rise of hybrid wars. 2007.

[18] Hornstein O. A look at one of the biggest years in quantum. UKTN. 2026. https://www.uktech.news/databyte/a-look-at-one-of-the-biggest-years-in-quantum-20260416.

[19] Inglesant P, Jirotka M, Hartswood M. Responsible Innovation in Quantum Technologies applied to Defence and National Security. 2018. p. 23.

[20] Inglesant P, Webb H, Ten Holter C, Patel M, Jirotka M. The responsible innovation of disruptive technologies. In: The SAGE handbook of digital society. 2023.

[21] Karsa A, Fletcher A, Spedalieri G, Pirandola S. Quantum illumination and quantum radar: a brief overview. Rep Prog Phys Phys Soc. 2024;87(9). 10.1088/1361-6633/ad6279. 10.1088/1361-6633/ad6279 39087757

[22] Keselman G, Murray F. Dual-Use Is a Strategy, Not a Category (Nor a Trap). War on the Rocks. 2025. https://warontherocks.com/2025/01/dual-use-is-a-strategy-not-a-category-nor-a-trap/.

[23] Krelina M. Military and security dimensions of quantum technologies: a primer. SIPRI; 2025. 10.55163/ZVTL1529.

[24] McKay E. “Keep the fight unfair”: military rhetoric in quantum technology. 2022. http://arxiv.org/abs/2203.01415.

[25] McKeran W. Fusion Doctrine: one Year on. 2019. https://www.rusi.org.

[26] National Research Council (US) Committee on Research Standards and Practices to Prevent the Destructive Application of Biotechnology. Biotechnology research in an age of terrorism. National Academies Press (US); 2004. http://www.ncbi.nlm.nih.gov/books/NBK222048/. 25057686

[27] NIST. PQC Standardization Process—Post-Quantum Cryptography | CSRC | CSRC. CSRC | NIST. 2025. https://csrc.nist.gov/projects/post-quantum-cryptography/post-quantum-cryptography-standardization.

[28] NPSA. Trusted Research Evaluation Framework. 2024. https://www.npsa.gov.uk/blog/trusted-research-evaluation-framework.

[29] OECD. An overview of national strategies and policies for quantum technologies. OECD Digital Economy Papers. 2025. 10.1787/5e55e7ab-en.

[30] Portillo V, Greenhalgh C, Craigon PJ, Ten Holter C. Responsible Research and Innovation (RRI) prompts and practice cards: a tool to support responsible practice. In: Proceedings of the first international symposium on trustworthy autonomous systems. 2023. p. 1–4. 10.1145/3597512.3599721.

[31] Pustovit SV, Williams ED. Philosophical aspects of dual use technologies. Sci Eng Ethics. 2010;16(1):17–31. 10.1007/s11948-008-9086-1. 18937057 10.1007/s11948-008-9086-1

[32] Responsible Technology Institute. Responsible quantum – from theory to practice. RTI. 2024. https://www.rti.ox.ac.uk/responsible-quantum-from-theory-to-practice/?utm_source=chatgpt.com.

[33] Schmidt A, Artaud A, Aydinoglu AU, Bötticher A, Bravo RA, Chiofalo M, Coates R, Ercan I, Grinbaum A, Haworth E, Ten Holter C, de Jong E, Karstens B, Kettemann MC, Knörr A, Lee CAL, Marco F, Mehnert W, Meyer JC, Seskir ZC, et al. Current and Future Directions for Responsible Quantum Technologies: a ResQT Community Perspective. 2025. 10.48550/arXiv.2509.19815. arXiv:2509.19815.

[34] Schnaufer T. Redefining Hybrid Warfare: Russia’s Non-linear War against the West. J Strateg Secur. 2017;10(1). 10.5038/1944-0472.10.1.1538.

[35] Scholten TL, Williams CJ, Moody D, Mosca M, Hurley W, Zeng WJ, Troyer M, Gambetta JM. Assessing the Benefits and Risks of Quantum Computers. 2024. 10.48550/arXiv.2401.16317. arXiv:2401.16317.

[36] Semiconductor Industry Association. Chipping Away: assessing and Addressing the Labor Market Gap Facing the U.S. Semiconductor Industry. 2023. http://www.semiconductors.org/chipping-away-assessing-and-addressing-the-labor-market-gap-facing-the-u-s-semiconductor-industry/.

[37] Seskir ZC, Umbrello S, Coenen C, Vermaas PE. Democratization of quantum technologies. Quantum Sci Technol. 2023;8(2):024005. 10.1088/2058-9565/acb6ae.

[38] Shelley-Egan C. The risk of ethicalisation in ethical engagement with quantum technologies: some brief considerations. NanoEthics. 2025;19(2):7. 10.1007/s11569-025-00471-2.

[39] Shor PW. Polynomial-time algorithms for prime factorization and discrete logarithms on a quantum computer. SIAM J Comput. 1996;26(5):1484–509. 10.1137/S0097539795293172.

[40] Stilgoe J, Owen R, Macnaghten P. Developing a framework for responsible innovation. Res Policy. 2013;42(9):1568–80. 10.1016/j.respol.2013.05.008.

[41] Taddeo M, Blanchard A, Pundyk K. Consider the ethical impacts of quantum technologies in defence now. Nature. 2024;634:779–81. 10.1038/d41586-024-03376-4. 39438750 10.1038/d41586-024-03376-4

[42] Technologies of War and Peace | Imperial News. Imperial College London. 2013. https://www.imperial.ac.uk/news/122899/technologies-war-peace/.

[43] Ten Holter C, Inglesant P, Jirotka M. Reading the road: challenges and opportunities on the path to responsible innovation in quantum computing. Technol Anal Strateg Manag. 2021. 10.1080/09537325.2021.1988070.

[44] Ten Holter C, Inglesant P, Pijselman M, Jirotka M. Towards responsible quantum computing; 2024.

[45] Ten Holter C, Inglesant P, Srivastava R, Jirotka M. Bridging the quantum divides: a chance to repair classic(al) mistakes? Quantum Sci Technol. 2022;7(4):044006. 10.1088/2058-9565/ac8db6.

[46] UKRI. Un-jammable quantum tech takes flight to boost UK’s resilience. 2024. https://www.ukri.org/news/un-jammable-quantum-tech-takes-flight-to-boost-uks-resilience/.

[47] US Congress Office of Technology Assessment. Technologies underlying weapons of mass destruction (OTA-BP-ISC-115). US Congress; 1993.

[48] Vermaas PE. The societal impact of the emerging quantum technologies: a renewed urgency to make quantum theory understandable. Ethics Inf Technol. 2017;19(4):241–6. 10.1007/s10676-017-9429-1.

[49] Vogiatzoglou P, van Hoboken J. The EU’s Digital Sovereignty and Quantum Technologies: to What End? (SSRN Scholarly Paper No. 5461374). Social Science Research Network. 2025. 10.2139/ssrn.5461374.

[50] von Schomberg R. A vision of responsible research and innovation. In: Owen R, Bessant J, Heintz M, editors. Responsible innovation. New York: Wiley; 2013. p. 51–74. http://onlinelibrary.wiley.com/doi/10.1002/9781118551424.ch3/summary.

[51] Walker-Munro B. Moving beyond “dual use”: quantum technologies and the need for new research security paradigms. EPJ Quantum Technol. 2025;12(1):136. 10.1140/epjqt/s40507-025-00448-w.

[52] Winner L. Do artifacts have politics? Daedalus. 1980;109(1):121–36.

[53] Zhang C. US Puts Export Controls on Quantum Computers. American Physical Society. APS. 2024. https://www.aps.org/apsnews/2024/10/export-quantum-computer.

